# Acceptability of genetically engineered algae biofuels in Europe: opinions of experts and stakeholders

**DOI:** 10.1186/s13068-020-01730-y

**Published:** 2020-05-22

**Authors:** Jessica Varela Villarreal, Cecilia Burgués, Christine Rösch

**Affiliations:** Institute for Technology Assessment and Systems Analysis (ITAS), Karlsruhe Institute of Technology (KIT), Karlstr.11, 76133 Karlsruhe, Germany

**Keywords:** Genetically modified organisms, Gene editing, Algae, Biofuel, Social perception, Acceptance, Risk perception, Survey

## Abstract

**Background:**

The development of alternative pathways for sustainable fuel production is a crucial task for politics, industry and research, since the current use of fossil fuels contributes to resource depletion and climate change. Microalgae are a promising option, but the technology readiness level (TRL) is low and cannot compete economically with fossil fuels. Novel genetic engineering technologies are being investigated to improve productivity and reduce the cost of harvesting products extracted from or excreted by microalgae for fuel production. However, high resource efficiency and low costs alone are no guarantee that algae fuels will find their way into the market. Technologies must be accepted by the public to become valuable for society. Despite strong efforts in algae research and development, as well as political commitments at different scales to promote algae biofuels for transport sectors, little is known about public acceptance of this alternative transport fuel. Despite the advantages of algae technology, genetically engineered (GE) microalgae can be controversial in Europe due to risk perception. Therefore, the aim of this study was to investigate, for the first time, the knowledge and views of European experts and stakeholders on the conditions and requirements for acceptability of GE microalgae for next generation biofuel production.

**Results:**

The results of the survey-based study indicate that the majority of the respondents believe that GE algae biofuels could provide strong benefits compared to other fuels. The majority would choose to be final consumers of engineered algae biofuels, if there is clear evidence of their benefits and open communication of potential risks. They believe that closed production systems with high security standards and rigorous risk assessment should be applied to avoid unintended impacts on humans and nature. Some respondents, however, are not convinced about the need to alter natural occurring algae strains to increase productivity, arguing that there is a huge unexplored variety, and that the consequences of using genome editing are still unknown.

**Conclusions:**

This evaluation of the opinions held by European experts and stakeholders regarding GE algae biofuels provides valuable and differentiated insights, both for future research and for the development of feasible socio-technical algae systems for next generation biofuel production. The identified conditions and requirements for achieving public acceptability can support the (re-)design of this innovative technology and adaptation of the framework conditions towards the implementation of algae biofuels in Europe.

## Background

Sustainable biofuel alternatives have been deeply investigated for decades in order to replace fossil fuels for future mobility [29]. The potential of using microalgae to produce biofuels continues to be investigated. Algae technology is economically unsustainable and can only contribute to mitigating climate change under certain conditions [28]. In terms of potential to reduce costs, the most important common factor is the increment of average productivity (yield) [24]. Although in recent years, some higher yields have been achieved by different cultivation strategies using natural algal strains such as *Tetraselmis suecica* and *Nannochloropsis oculata* [33, 38], algal biofuels still cannot economically compete with fossil fuels [3, 10]. Ketzer et al. [13] concluded in their review that a higher energy return of investment (EROI) could be achieved, from a biological point of view, by enhancing the efficiency of photo-conversion, which would lead to higher biomass and energy yields. The research focus is currently therefore to increase and modify the accumulation or release of energy products or their precursors (e.g., lipids, alcohols, hydrocarbons) in photosynthetic microalgae through genetic engineering. Although the application of genetic engineering to improve energy production phenotypes in eukaryotic microalgae is in its infancy, significant advances in the development of genetic manipulation tools have been achieved recently with microalgal model systems, and are being used to manipulate central carbon metabolism in these organisms [26]. It is likely that many of these advances can be extended to industrially relevant organisms, and that this will be a major research advance concerning the commercialization of algae biofuels [7, 10].

Precise CRISPR/Cas9-based genome editing of industrial algal strains such as *Nannochloropsis*, which accumulates oil as a source of plant-like oils for biofuel production during nitrogen deprivation, have been conducted by Wang et al. [34], opening opportunities for microalgae-based biotechnological applications. Metabolic engineering of *Chlamydomonas reinhardtii* was presented as an option to be optimized for biofuel production, due to the achievement of higher yields of terpenoids [36]. Recently, a joint study also pointed *Chlamydomonas reinhardtii* as the next chassis for sustainable synthetic biology [6]. Furthermore, protein engineering has been recently used to enhance isobutanol production in the unicellular cyanobacterial strain *Synechocystis* PCC 6803 [21, 19, 20, 37].

The use of GE microalgae strains for the release of biofuel precursors to the culture broth for direct separation without cell harvesting has been thoroughly investigated in the Photofuel project (http://www.photofuel.eu). Metabolic engineering strategies were employed by Liu, Miao et al. [20] to generate 1-butanol producing *Synechocystis*. After the selection of enzymes and promoters, 836 mg L^−1^ of 1-butanol were produced in a flask. By optimizing the cultivation condition, an in-flask titer of 2.1 g L^−1^ and a maximal cumulative titer of 4.7 g L^−1^ were observed in the long-term cultivation. These strains with enhanced or modified metabolic activity show great potential for biotechnological exploitation. Since there is a highly controversial general debate around agricultural genetic engineering in Europe [5, 17], it cannot be ruled out that there might be similar concerns about the impact of GE microalgae on the environment and human health. Whether such a debate will arise on the topic of algae, and how this is addressed, will play a key role in implementation and commercialization of engineered microalgae, including their application for biofuel production [4].

The present study was conducted within the European Union (EU) H2020 project Photofuel, in order to investigate the conditions and requirements for the implementation of a novel technology for engineered microalgae biofuel production. The objective of the work was to gain insights into the opinions and attitudes of European experts and stakeholders regarding their knowledge, perception and views of this technology as well as on their conception regarding its public acceptability.

## Results

The survey scored 130 valid responses from across the EU on 16 different questions.

### Descriptive statistical analysis

#### 1. Sociodemographic profile

**Fig. 1:**
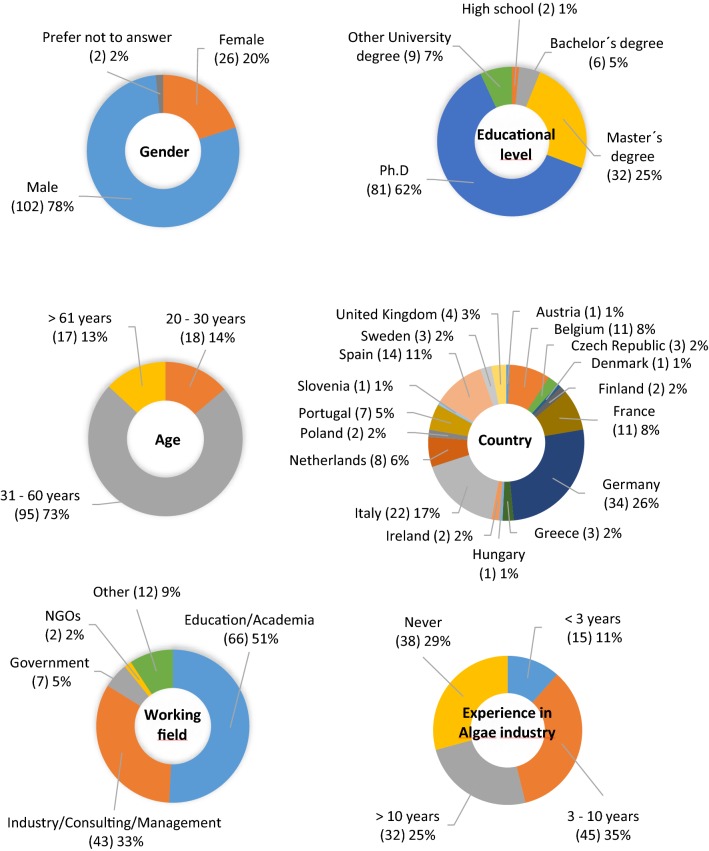
Sociodemographic data (absolute results are shown in brackets)

The sociodemographic profile of the respondents (Fig. 1) shows a high response rate from males (78%). Respondents had a high educational level; 62% had a Ph.D., and only 6% did not have a University degree. A high number of respondents had experience in the algae industry (71%). Most worked in education or academia (51%), followed by industry, consulting or management (33%). The majority of respondents (73%) were between 31 and 61 years. Answers from 17 of the 27 EU countries and from the former EU country United Kingdom, were recorded. Most of the respondents were from Germany (26%), followed by Italy (17%), Spain (11%), France (8%), Belgium (8%), the Netherlands (6%) and Portugal (5%). A low percentage (between 3% and 1%) of respondents were from the United Kingdom, Sweden, Poland, Ireland, Greece, Finland, Czech Republic, Austria, Slovenia, Hungary and Denmark. No response was obtained from Bulgaria, Croatia, Cyprus, Estonia, Latvia, Lithuania, Luxembourg, Malta, Romania and Slovakia.

Plots from RStudio (not shown) exposed two main groups: the most noticeable group belonged to the field of education or academia, in the age group of 31 to 60 years; the second group belonged to industry, consulting or management, and to the same age group. In both groups, most respondents were males, although the number of females was higher in the education or academia group.

#### 2. Perceptions of expected benefits and risks of GE algae biofuel

**Fig. 2:**
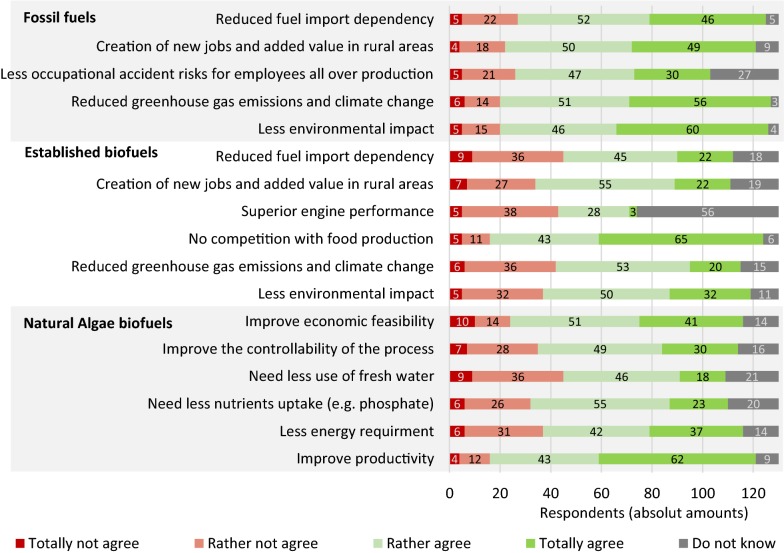
Perceptions of respondents about expected benefits of GE algae biofuel among fossil fuels, established biofuels and natural algae biofuels

Most respondents perceived that the expected benefits of GE algae biofuel were high, in contrast to fossil fuels, established biofuels and even to natural algae biofuels (Fig. 2). They were noticeably higher when compared to fossil fuels, especially in the options that referred to environmental issues. In the case of the expected benefits of GE algae biofuel among established biofuels, the highest agreement level was for “No competition with food production”. The lowest was for “Superior engine performances”, but in this case the highest amount of “Do not knows” was also observed. The expected benefits of GE algae instead of natural strains are also significant, “Improvement of economic feasibility” and “The improvement of productivity” being the most supported options.

When respondents were asked about choosing to replace fossil fuels, at least partially, with GE algae biofuel in order to use fewer limited resources and reduce climate change, their answer tended to be positive (31% totally agreed, and 40% rather agreed). This indicates that partially replacing fossil fuels with GE algae biofuel could be a positive option to mitigate climate change.

**Fig. 3:**
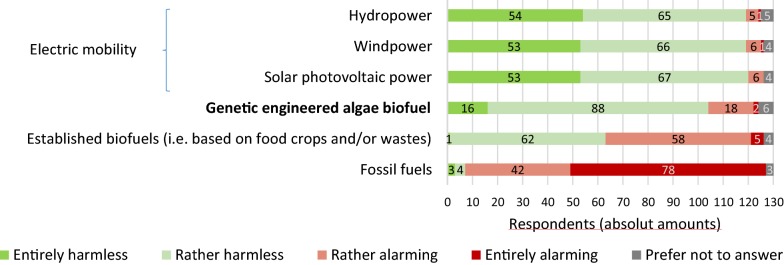
Perceptions of respondents about general risks of future mobility fuels or power sources

Considering the perception of the general risks (i.e., health, environment and accidents) of different fuels and power sources that could be used for future mobility (Fig. 3), 92% of the respondents indicated fossil fuels as the most alarming case, followed by established biofuels (48%). Most of the respondents (92%) considered wind power, hydropower and solar photovoltaic power as the most harmless options, followed by 80% of the respondents who believe that GE algae biofuel would also be a harmless alternative. Higher amounts of “Do not knows” were observed for GE algae biofuel (5%) and Hydropower (4%), indicating that people are less informed about these topics.

#### 3. Perceptions of social acceptance of GE algae biofuel

**Fig. 4:**
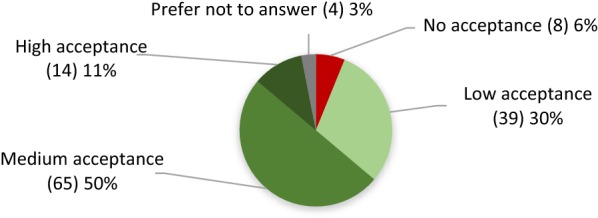
Opinions of respondents about general social acceptance of GE algae biofuel in the EU (absolute amounts are in brackets)

Respondents believe that GE algae biofuel will have a medium (50%) to high (11%) general acceptance in the EU (Fig. 4), although a relatively high percentage think the opposite.

**Fig. 5:**
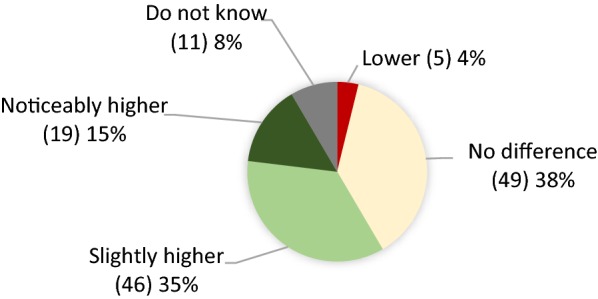
Opinions of respondents about the improvement of acceptance of GE algae biofuel due to the use of gene-editing techniques (absolute amounts are in brackets)

When asking if this acceptance would change with the use of novel precise gene-editing techniques instead of traditional genome modification techniques, an average perception between no difference (38%) and a slightly higher acceptance (35%) was obtained (Fig. 5).

**Fig. 6:**
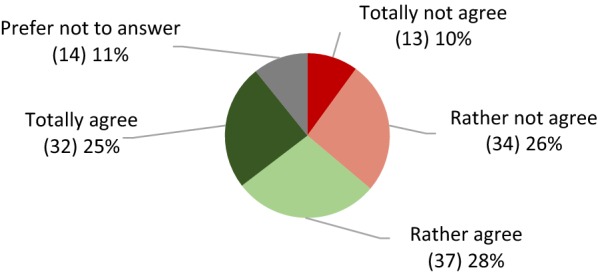
Opinions of respondents about not regulating gene-editing as GMO (absolute amounts are in brackets)

Respondents were asked if they thought gene-editing should fall under current GMO regulation. A clear difference of opinions was observed (Fig. 6), although 53% gave a positive answer, 36% gave a negative answer and 11% did not know. This question had the most “Do not knows” within the social acceptance section of the questionnaire.

#### 4. Personal attitudes as consumers

**Fig. 7:**
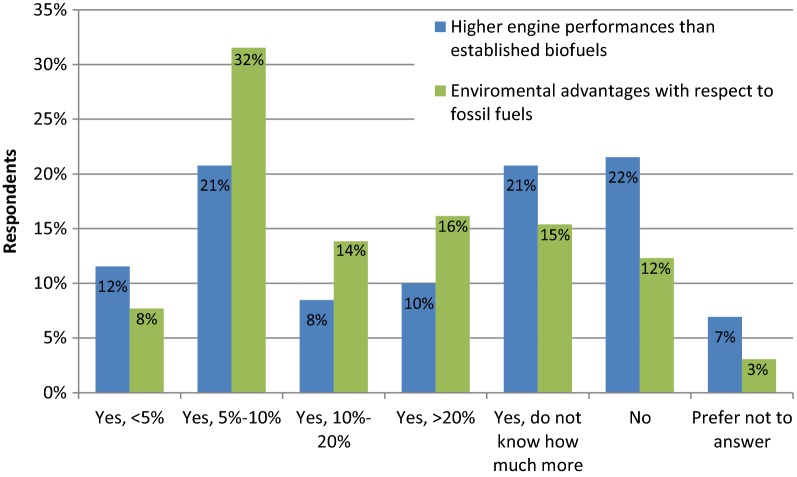
Willingness to spend more money on GE algae biofuel if higher engine performances than those for established biofuels (blue) were achieved, and if environmental advantages compared to fossil fuels (green) were attained

The majority of the respondents (72%) would choose to be final consumers of GE algae biofuel, while 18% did not know, and 10% answered negatively. Their willingness to spend more money on GE algae biofuel if higher engine performances compared to those of established biofuels were achieved, and in cases where more environmental advantages were achieved compared to fossil fuels are shown in Fig. 7. If GE algae biofuel could achieve higher engine performances, then 21% of respondents answered that they were willing to pay 5-10% more money; but the same percentage answered that they were not prepared to spend more money. Finally the same percentage answered that they did not know how much more money they would spend. In cases where biofuel had environmental advantages compared to fossil fuels, the highest percentage of respondents (32%) answered that they were willing to spend 5–10% more money. When compared with the previous question, significantly fewer respondents answered negatively, and there were fewer respondents who did not know how much more money they would be willing to spend. In general, for the higher ranges of money to be spent, it seems people are more interested in environmental care than in getting better engine performances.

#### 5. Individual suggestions

**Fig. 8:**
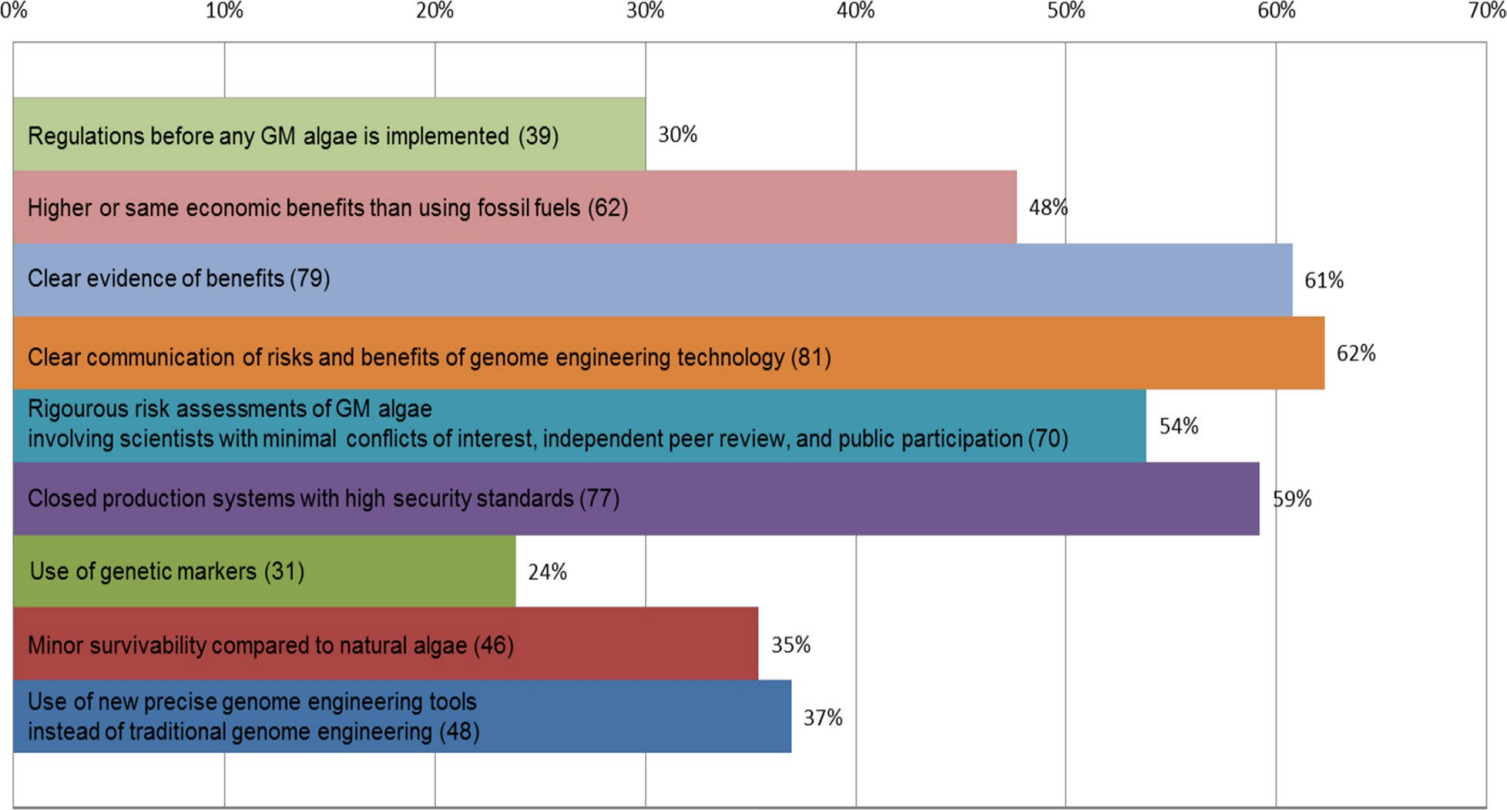
Suggestions of the respondents on how to improve social acceptance of GE algae biofuel

Respondents were asked how to improve social acceptance of GE algae biofuel (Fig. 8). The most selected options were to clearly communicate the risks and benefits of genome engineering technology (62%), to have clear evidence of benefits (61%), and to use closed production systems with high security standards (59%). The second place options were to carry out rigorous risk assessments of genetically modified (GM) algae, involving scientists with minimal conflicts of interest, independent peer review, and public participation (54%), and to achieve higher or equal economic benefits than using fossil fuels (48%). The options with lowest interest were the use of genetic markers in order to identify the presence of GE algae as well as the flow of a particular genome engineered trait, if released into the environment (30%), and the necessity for regulations before any genome engineered species is released (24%). However, the percentages for these options were not low.

### Inductive statistical analysis

**Fig. 9:**
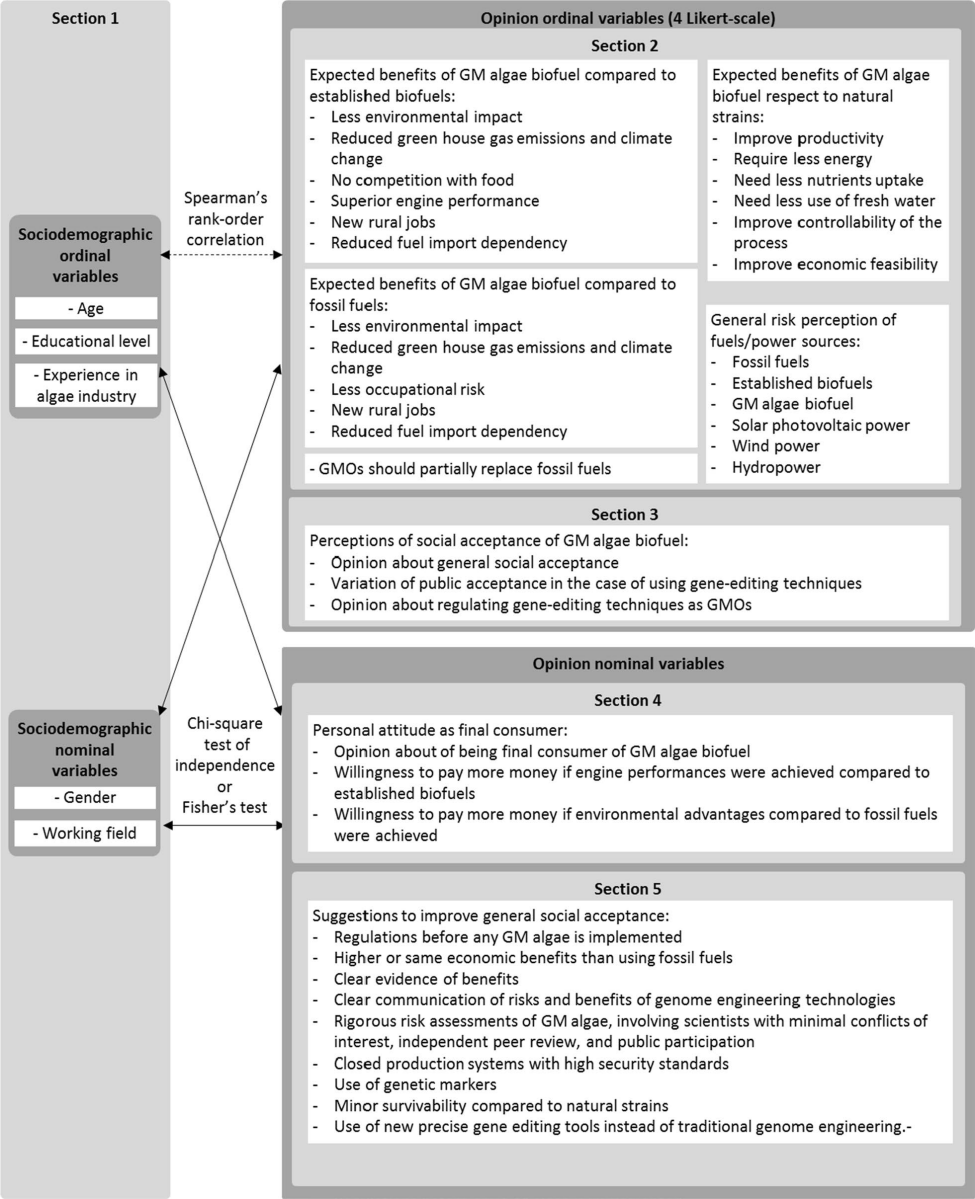
Summary and overview of variables and the statistic tests performed to find statistical relationships

Inductive statistical tests were done to seek possible relationships between the variables, as shown in Fig. 9. Only statistically significant results (*p* values < 0.05) are shown in Tables 1, 2 and 3.

**Table 1:** Correlation coefficients and *p* values (only *p* values < 0.05 are shown) after Spearman’s rank order between sociodemographic ordinal variables and opinion ordinal variables

Sociodemographic ordinal variables	Opinion ordinal variables	Spearman’s rank order coefficient	*p* value
Age	*Expected benefits of GE algae biofuel compared to established biofuels*		
	Less environmental impact	− 0.17	0.025
	Reduced GHG emissions and climate change	− 0.19	0.023
Educational level	*Expected benefits of GE algae biofuel compared to established biofuels*		
	Less environmental impact	0.02	0.044
	Reduced GHG emissions and climate change	− 0.03	0.001
	New rural jobs	0.06	0.040
	*Expected benefits of GE algae biofuel compared to natural strains*		
	Require less energy	− 0.03	0.021

**Table 2:** Chi-square test correlations (*p* values < 0.05) and respective Cramer’s V values between sociodemographic ordinal variables and opinion nominal variables

Sociodemographic ordinal variables	Opinion nominal variables	*χ*^2^-test (*p* value)	Cramer’s V
Age	*Suggestions to improve general social acceptance*		
	Closed production systems with high security standards	0.041	0.221
	Use of new precise gene editing tools instead of traditional genome engineering	0.044	0.220
Educational level	*Personal attitude as final consumer*		
	Opinion about being final consumer of GE algae biofuel	0.037	0.251
	*Suggestions to improve general social acceptance*		
	Clear communication of risks and benefits of genome engineering technologies	0.004	0.346
Experience in algae industry	*Personal attitude as final consumer*		
	Opinion about being final consumer of GE algae biofuel	0.024	0.236

**Table 3:** Chi-square test correlations (*p* values < 0.05) and respective Cramer’s V values between sociodemographic nominal variables and opinion nominal variables

Sociodemographic nominal variables	Opinion nominal variables	χ2-test (*p* value)	Cramer’s V
Gender	*General risk perception of fuels/power sources*		
	Hydropower	0.034	0.285
Working field	*Expected benefits of GE algae biofuel compared to established biofuels*		
	No competition with food	0.011	0.246
	*Expected benefits of GE algae biofuel compared to natural strains*		
	Require less energy	0.016	0.242
	Improve the controllability of the process	0.050	0.225
	*Perceptions of social acceptance of GE algae biofuel*		
	Opinion about regulating gene-edited organisms as GMOs	0.024	0.236
	*General risk perception of fuels/power sources*		
	Fossil fuels	0.022	0.237
	Wind power	0.002	0.267

Table 1 shows Spearman’s rank correlation coefficients between sociodemographic ordinal variables and opinion ordinal variables. Although some *p* values lower than 0.05 were found, indicating that the relationships are statistically significant, none of the Spearman correlation coefficients had high values, indicating weak relationships between the variables. These weak relationships were the following:

1. The younger the respondents the more they agreed that the expected benefits of GE algae biofuel, compared to established biofuels, include less environmental impact and reduced greenhouse gas emissions and climate change.
2. People with a higher educational level agreed more that the expected benefits of GE algae biofuel, compared to established biofuels, include less environmental impact and new rural jobs.
3. People with a higher educational level were more skeptical that the expected benefits of GE algae biofuel, compared to established biofuels, include reduced greenhouse gas emissions; and, compared to natural strains, a reduced energy demand.

Table 2 shows only *p* values < 0.05 after Chi-square test was done between sociodemographic ordinal variables and opinion nominal variables, and their respective Cramer’s V values. Following the rule for the interpretation of Cramer’s V values, the following moderate relationships were found:

1. Respondents older than 31 years seemed to have a higher acceptance of using new precise genome editing tools instead of traditional genome engineering.
2. Respondents younger than 30 and older than 60 years (approx. 77%) believed that the use of closed production systems with high security standards should be a priority.
3. A tendency was observed, where the higher the educational level of respondents was, the higher willingness they had to be final GE algae biofuel consumers, but also the number of respondents who answered ‘Do not know’ to this question increased with educational level.
4. Respondents who had never been active in the algae industry and respondents with more than 10 years of experience in the algae industry showed a lower tendency for willingness to be final GE algae biofuel consumers. The main difference between these two groups was that the respondents who had never been active in the algae industry also had a greater percentage that replied, ‘Do not know’.

The only strong relationship was the following:

- Respondents with a higher educational level gave more importance to the clear communication of risks and benefits of genome engineering technology.

Table 3 shows only *p* values < 0.05 after Chi-square test was done between sociodemographic nominal variables and opinion ordinal variables, and their respective Cramer’s V values. Following the rule for the interpretation of Cramer’s V values, the following moderate relationships were found:

1. Although most female respondents believe that hydropower is rather harmless, they are cautious in affirming that hydropower is an entirely harmless source of power.
2. Although most of the respondents from every professional field had a higher tendency to believe that one of the benefits of GE algae biofuel compared to fossil fuels is the lack of competition with food production, the group of respondents working in education or academia had the highest tendency for agreement (37.9% rather agreed, and 53% totally agreed).
3. Most respondents tended to agree that one of the benefits of using GE algae biofuel compared to natural strains would be the requirement for less energy in its production, but in the group of respondents working in education or academia there were more respondents that did not know, (14.6%) while the respondents working in industry were more skeptical of this benefit (2.3% totally not agreed and 41.9% rather not agreed).
4. Most respondents tended to agree in affirming that one of the benefits of using GE algae biofuel compared to natural strains would be improved controllability of the process. The group of respondents working in education or academia agreed more (40.9% rather agreed and 25.8% totally agreed), while the group of respondents working in industry had a higher percentage that did not know (14%).
5. Most respondents working in education or academia, and also in industry, tended to agree in affirming that organisms with small genetic changes achieved by gene-editing techniques, should not fall under the current regulations for GMOs. 57.1% of respondents working for the government did not know.
6. Although more than 90% of the respondents believe that the general risk of using fossil fuels is alarming, some respondents working in education and academia were skeptical about this with 4.5% who answered that it was rather harmless, and 4.5% that answered it was entirely harmless. While 100% of respondents working in the government agreed that this source of power is alarming (rather alarming 14.3%, and entirely alarming 85.7%).
7. Although most respondents believe that wind power is harmless (more than 88%), 7% of the respondents working in industry rated this energy source as harmful (4.7% rather alarming and 2.3% entirely alarming). While 100% of respondents working in the government agreed that this source of power is harmless (rather harmless 57.1%, and entirely alarming 42.9%).

## Discussion

First and second-generation biofuels cannot meet global demands in a sustainable way [1]. Therefore, third generation biofuels produced with microalgae are considered to play a crucial role in achieving long-term climate policy objectives in the mobility sector. However, the production of algal fuel is not yet economically feasible nor sustainable regarding the demand of energy and the release of greenhouse gas emissions [13, 28]. Research and development is trying to overcome the techno-economic and ecological obstacles that hinder the implementation of algae biofuel production for sustainable mobility. New methods of genetic engineering, such as genome editing, can foster the achievement of this objective by increasing algae productivities and yields and by facilitating the release of fuels or fuel precursors into the cultivation media to make the process and respective fuel harvesting more efficient. Scientists have discovered new ways of using GE yeast for biofuel production, making yeasts more tolerant to the self-produced ethanol [15].

In the EU, research with GE algae is restricted to authorized laboratories and pilot plants, which need safeguard precautions to avoid any risks that could eventually result from the uncontrolled release of these GE algae into the environment. Since the process of producing fuels with GE algae is at a low TRL level, there is no information available about the perception of GE algae by experts and stakeholders. Our results indicate that there is no evidence about possible concerns or even opposition to the technology. This could be related to the fact that the media have not reported on it and knowledge about GE algae is not yet widespread. Another reason could be that the use of GE algae for biofuel production would be to replace the unpopular first generation biofuels. There is evidence that in contrast to GE applied in food production, there are no such concerns for GE crops, which are used to produce first generation bioethanol and biodiesel from starch (e.g., corn) and vegetable oil feedstock (e.g., soybean) [32]. Particularly, North and South American countries are large-scale producers of GE corn and soya that are not only used for food and feed, but also for fuel production. Moreover, research on GE is ongoing for second-generation bioethanol production from cellulosic biomass, which is both abundant and renewable, and a promising alternative to bioethanol produced with food crops. Plant genetic engineering promises to have a key role in decreasing biofuel production costs by deconstructing plant cell-wall polysaccharides by higher levels of cellulases and hemicellulases, suppressing lignin biosynthesis enzymes, which reduce the need for pretreatment, or by increasing the content of polysaccharides or the overall plant biomass [31].

Although our results indicate a higher preference for GE algae biofuel compared to first generation biofuels, it cannot be concluded that people will purchase the product once algae biofuel is on the market, and even pay more money for it, compared to other fuels. Since there is an intensive debate on sustainable mobility in general and a trend to ban cars with combustion engines, it is not surprising that mobility provided by green electricity based on hydro, wind and solar power is regarded as even more desirable due to lack of emissions and climate-friendliness. The results from this survey are compatible with the findings of Moula et al. [23] and Kubik [14]. Moula et al. [23] found that only 60% of respondents are willing to switch towards purchasing second-generation biofuels, and that car owners responding to the question about the ideal fuel would prefer electricity (60%) over hydrogen (20%) and hybrid (20%). Kubik [14] found that when asked to choose among ethanol, hydrogen and electricity, the respondents of a US National Renewable Energy Laboratory survey rated ethanol as the worst fuel to be used in personal vehicles once gasoline is no longer available. Respondents tended to have environmental concerns as their primary motivation. Data suggests that the American public is largely unaware of biofuels, being an important finding that has been used to explain the sometimes reported low levels of support [35]. As Einsiedel and Eastlick [8] reported, citizens do not exclusively rely upon knowledge when forming opinions about political and scientific issues. Instead, individuals will often rely on heuristic shortcuts to make sense of complex and controversial issues.

Adeniyi et al. [1] found that fast-track algae biofuel production could be a feasible midterm solution to replace fossil transportation fuels in trucks and airplanes which will not be fueled by renewable electricity in the next 10 to 20 years. Moreover, fuel blends with algal biofuel give positive results on combustion and emission (www.photofuel.eu). The opinion of the experts and stakeholders in our study support the statement of Adeniyi et al. [1]. A high fraction of experts (71%) expressed that a partial replacement of fossil fuels with GE algae biofuel could be a positive option to mitigate climate change. A sustainable future of mobility should not be viewed as the end of the internal combustion engine, currently the main source of vehicular propulsion [18]. The development and use of more sustainable and environmentally friendlier options, like GE algae biofuel, should therefore be considered for the transformation phase.

In the EU, the use of GE crops and GMOs in agriculture is subject to extensive restrictions since public opposition to GM technology is widespread [9]. There is also opposition in the United States. In a representative survey of U.S. residents, 64% opposed GM, and 71% of GM opponents (45% of the entire sample) were “absolutely” opposed—that is, they agreed that GM should be prohibited no matter the risks and benefits. These absolute opponents predicted support for legal restrictions on GE foods, even after controlling for explicit risk–benefit assessments. This research suggests that many opponents are evidence-insensitive and will not be influenced by arguments about risks and benefits [30].

Nowadays, the production of biofuels with GE algae still has not been a focus of public opinion, and no significant negative media reports or public opinions have been found. But if large-scale production of algal fuel was implemented, it is not unlikely that opposition could arise due to the general controversial debate on genome editing in plant breeding and microorganisms. Since no method of genetic modification is without the possibility of unintended effects, genetic engineering in general and the new technique of genome editing are likely to be subject to the same underlying factors of information processing and risk perception by the public, media and individuals that have been found across multiple other emerging technologies. Most of our survey respondents have an academic background, and it is possible that the results would be different for the general public.

If a technology is merely perceived as similar to the incumbent one, consumers will not be motivated to adopt it. This is especially true when an innovation is more expensive than the preceding technology [27]. Our results reveal respondents’ expectations that GE algae biofuels could provide strong benefits among other fuels, mainly due to the reduction of environmental impacts in general, and climate change and land use competition. However, this expectation cannot be met by science today. The same is true for the statement that GE algae are superior to natural strains and can improve the environmental compatibility and economic viability of algae biofuels. There is evidence at lab and pilot scale about their superiority and lack of significant disadvantages, but this has to be proven at a commercial scale, since this is required for the genetic stability of GE algae.

Even if algae can keep the promises of providing additional benefits to humans and nature, this does not necessarily lead to public acceptance. Even crops with great potential to combat major health problems due to malnutrition, such as GE rice with high contents of vitamin A, are not accepted by the public. GE opponents have strongly resisted programs to provide subsistence farmers in Africa and Asia with GE “golden rice” that produces vitamin A precursor beta-carotene [11]. Our results clearly indicated that although most respondents generally would accept the use of GE algae for fuel production, some were concerned about the potential drawbacks and potential risks for nature. In contrast to their own more positive perception, many respondents consider the acceptance of the public to be much lower since they expect that there will be a significant share of people with low or no acceptance at all, mainly due to genetic engineering.

Our results do not indicate a significant difference in perception between genome editing and other techniques to modify microorganisms. Most respondents are not convinced that new techniques of genome editing significant increase acceptance for GE algae biofuel in the public. However, many of them are not aware of, and do not fully understand, genome editing and the differences in technology well enough to judge on this topic.

Some respondents are not convinced about the need to alter natural occurring algae strains to increase productivity since there is still a wide variety of natural algae strains to explore, and because the consequences of genome editing are unknown. Despite these concerns, the majority of the respondents would choose to be final consumers of GE algae biofuel.

The results of the survey indicate that, if conducive social and regulatory conditions are in place, it can substantially increase the positive impacts of GE algae biofuels on human welfare and sustainability. However more decisive benefits are required in order to convince the consumer to adopt algae biofuels, given their current high costs.

## Conclusions

There are numerous challenges in realizing the potential of algae biofuels envisioned by many policy-makers. The technical challenges to improve the sustainability of algae biofuel production to replace a significant fraction of transportation fuel have been well described. The use of genetic engineering can potentially address many of these technical challenges and environmental concerns, but brings significant regulatory hurdles that have not been discussed extensively in the scientific community. Additionally, concerns about and even rejection of algae biofuel could hamper market entrance if algae fuel is not able to keep the promises made. However, alongside the development of GE algae, social acceptance issues have been underestimated. While social acceptance can emerge as a powerful barrier for algae biofuel development, our results provide insights into their social acceptability. The results of the survey show how experts interpret the use of GE algae for the production of biofuels, and the values, beliefs and expectations that guide those interpretations, as well as the hopes and intentions interlinked with those fuels. Our findings show that gaining insights into the opinions of experts and stakeholders towards GE algae can contribute to developing feasible socio-technical algae systems for biofuel production and to (re-) design the processes and adapt the framework conditions towards a higher acceptability of GE microalgae. While this research is a helpful step in gathering an understanding of public attitudes toward genetically engineered algal biofuels, future research will need to examine a number of key issues in order to arrive at a more nuanced understanding of opinion formation for the algal biofuels issue.

## Methods

### Questionnaire design and data collection

Based on a literature review and interviews with experts on genetic engineering of microorganisms, we drafted the structure and content of a survey to be conducted online in order to facilitate the participation of people in European countries. A long list of European experts in the fields of microalgae, biofuels, genetics and environment, as well as of stakeholders, such as non-governmental organizations (NGOs), was compiled and reviewed, using the report on stakeholders produced in the project “Algae and aquatic biomass for a sustainable production of 2nd generation biofuels—*AquaFUELs”* as the main source. A pre-test of the survey was conducted to verify the suitability of the questionnaire regarding its structure, comprehensibility and length. The online survey with the web-based questionnaire was performed using the platform www.soscisurvey.de. The experts and stakeholders were invited to participate via e-mail. In order to reach further experts and stakeholders and to increase the number of respondents, these persons were asked to forward the questionnaire to other experts and stakeholders from their fields of interest according to the snowballing approach discussed by Almeida et al. [2]. Further participants were recruited via professional business and research networks. The survey was conducted between September and November 2017.

The questionnaire comprised 16 (mainly closed) questions, which were structured into the following five sections.

1. Sociodemographic profile*: gender, age, educational level, country of residence, experience in the algae industry and respective professional field.*
2. *Perceptions of expected benefits and risks of GE algae biofuel:* opinions on the expected benefits of GE algae biofuel compared to fossil fuels, established biofuels and natural algae biofuels, as well as opinions on general risks of power sources used for mobility of the future.
3. *Perceptions of social acceptance of GE algae biofuel:* opinions about general social acceptance of GE algae biofuel in the EU, perceptions of how new gene-editing techniques might improve public acceptance compared to classical genetic engineering techniques, and opinions about classifying and regulating gene-editing techniques as genetically modified organisms (GMOs).
4. *Personal attitudes as consumers:* attitudes towards becoming potential final consumers of GE algae biofuel, as well as the willingness to pay more money in cases of advantages regarding higher engine performances compared to established biofuels, or environmental advantages compared to fossil fuels.
5. *Individual suggestions:* opinions about how public acceptance of GE algae biofuel could be improved.

### Data analysis

Data analysis was undertaken in two steps: (i) descriptive statistical analysis, and (ii) inductive statistical analysis. Descriptive statistical analysis was performed and presented by using Microsoft Excel^®^ [22] and RStudio [25]. Inductive statistical analysis was performed by using IBM-SPSS.25 [12]. Figure 9 summarizes and gives an overview of the variables and the statistical tests performed to find relationships between them. All opinion ordinal variables were ranked in a 4 Point Likert scale [16].

Correlations between sociodemographic ordinal variables and opinion nominal variables were completed by using Spearman’s rank correlation coefficient with SPSS software. The correlation coefficient can range in value from −1 to +1. The larger the absolute value of the coefficient, the stronger the relationship between the variables. Due to the lack of a significant number of respondents from some countries, no inductive analysis was done for this variable; therefore no country-specific results are shown in this paper.

Correlations between sociodemographic variables and opinion nominal variables were done by using the Chi-square test of independence with SPSS software. Chi-square test shows if there is a significant relationship between variables, but it does not say how significant and important this is. Cramer’s V is a post-test to give this additional information. In the cases where *p* values obtained from Chi-tests were lower than 0.05 additional Cramer’s V test was done in order to see the strength of the relationships. Cramer’s V values were interpreted where: values < 0.10 indicate weak relationships; values between 0.10 and 0.30 indicate moderate relationships, and values > 0.30 indicate strong relationships. Fisher’s test was done in cases of having two dichotomous categorical variables.

## Data Availability

All data generated or analyzed during this study are included in this published article.

## Appendices

### Appendix A

Tables 4, 5, 6 and 7.

**Table 4:** Sociodemographic ordinal variables

Variable name	Answers
Age	< 20 years 20–30 years 31–60 years > 61 years
Educational level	Did not complete high school High school Bachelor’s degree Master’s degree Other university degree PhD
Experience in algae industry	Never < 3 years 3–10 years > 10 years

**Table 5:** Opinion ordinal variables (4 Likert scale)

Variable name	Answers
*Expected benefits of GE algae biofuel compared to established biofuels*	
Less environmental impact	Totally not agree Rather not agree Rather agree Totally agree Do not know
Reduced greenhouse gas emissions and climate change	
No competition with food	
Superior engine performance	
New rural jobs	
Reduced fuel import dependency	
*Expected benefits of GE algae biofuel compared to fossil fuels*	
Less environmental impact	Totally not agree Rather not agree Rather agree Totally agree Do not know
Reduced greenhouse gas emissions and climate change	
Less occupational risk	
New rural jobs	
Reduced fuel import dependency	
*Expected benefits of GE algae biofuel compared to natural strains*	
Improve productivity	Totally not agree Rather not agree Rather agree Totally agree Do not know
Require less energy	
Need less nutrients uptake	
Need less use of fresh water	
Improve the controllability of the process	
Improve economic feasibility	
GMOs should partially replace fossil fuels	Totally not agree Rather not agree Rather agree Totally agree Do not know
*General risk perception of fuels/power sources*	
Fossil fuels	Entirely harmless Rather harmless Rather alarming Entirely alarming Prefer not to answer
Established biofuels	
*GE algae biofuel*	
Solar photovoltaic power	
Wind power	
Hydropower	
*Perceptions of social acceptance of GE algae biofuel*	
Opinion about general social acceptance	No acceptance Low Medium High acceptance Prefer not to answer
Variation of public acceptance in case of using gene-editing techniques	Lower No difference Slightly higher Noticeably higher Do not know
Opinion about regulating gene-edited organisms as GMOs	Totally not agree Rather not agree Rather agree Totally agree Prefer not to answer

**Table 6:** Sociodemographic nominal variables

Variable name	Answers
Gender	Female Male
Land	Austria; Belgium; Bulgaria; Croatia; Cyprus; Czech Republic; Denmark; Estonia; Finland; France; Germany; Greece; Hungary; Ireland; Italy; Latvia; Lithuania; Luxembourg; Malta; Netherlands; Poland; Portugal; Romania; Slovakia; Slovenia; Spain; Sweden; United Kingdom
Working field	Education/academia Industry/consulting/management Government Non-governmental organization Journalism Other

**Table 7:** Opinion nominal variables

Variable name	Answers
*Personal attitude as final consumer*	
Opinion about being final consumer of GE algae biofuel	Yes No Do not know
Willingness to pay more money if higher engine performances were achieved compared to established biofuels	Yes, < 5% Yes, 5–10% more Yes, 10–20% more Yes, > 20% Yes, do not know how much more No
Willingness to pay more money if environmental advantages were achieved compared to fossil fuels	
*Suggestions to improve general social acceptance*	
Regulations before any genome engineered species is implemented	Yes No
Higher or same economic benefits than using fossil fuels	
Clear evidence of benefits	
Clear communication of risks and benefits of genome engineering technologies	
Rigorous risk assessments of GM algae, involving scientists with minimal conflicts of interest, independent peer review, and public participation	
Closed production systems with high security standards	
Use of genetic markers	
Minor survivability compared to natural strains	
Use of new precise gene editing tools instead of traditional genome engineering	

### Appendix B

Tables 8, 9, 10 and 11.

**Table 8:** *p* values after Spearman’s rank order correlation coefficients between sociodemographic ordinal variables (Appendix A: Table 4) and opinion ordinal variables (Appendix A: Table 5)

Sociodemographic ordinal variables	Opinion ordinal variables	Spearman’s rank order coefficient	*p* value
Age	*Expected benefits of GE algae biofuel compared to established biofuels*		
	Less environmental impact	− *0.17*	*0.025*
	Reduced GHG emissions and climate change	− *0.19*	*0.023*
	No competition with food	− 0.02	0.915
	Superior engine performance	− 0.09	0.268
	New rural jobs	− 0.13	0.389
	Reduced fuel import dependency	0.03	0.637
	*Expected benefits of GE algae biofuel compared to fossil fuels*		
	Less environmental impact	− 0.05	0.345
	Reduced GHG emissions and climate change	− 0.05	0.391
	Less occupational risk	− 0.25	0.326
	New rural jobs	− 0.06	0.807
	Reduced fuel import dependency	− 0.02	0.949
	*Expected benefits of GE algae biofuel compared to natural strains*		
	Improve productivity	− 0.23	0.148
	Require less energy	− 0.23	0.109
	Need less nutrients uptake	− 0.18	0.326
	Need less use of fresh water	− 0.08	0.902
	Improve the controllability of the process	− 0.2	0.355
	Improve economic feasibility	− 0.19	0.554
	GMOs should partially replace fossil fuels	− 0.02	0.255
	*Perceptions of social acceptance of GE algae biofuel*		
	Opinion about general social acceptance	− 0.15	0.167
	Variation of public acceptance in case of using gene-editing techniques	− 0.04	0.662
	Opinion about regulating gene-edited organisms as GMOs	0.09	0.665
	*General risk perception of fuels/power sources*		
	Fossil fuels	0.06	0.528
	Established biofuels	0.01	0.858
	GE algae biofuel	0.06	0.972
	Solar photovoltaic power	0.06	0.851
	Wind power	0.07	0.907
	Hydropower	− 0.02	0.897
Educational level	*Expected benefits of GE algae biofuel compared to established biofuels*		
	Less environmental impact	*0.02*	*0.044*
	Reduced GHG emissions and climate change	− *0.03*	*0.001*
	No competition with food	0.12	0.058
	Superior engine performance	− 0.04	0.314
	New rural jobs	*0.06*	*0.040*
	Reduced fuel import dependency	− 0.02	0.126
	*Expected benefits of GE algae biofuel compared to fossil fuels*		
	Less environmental impact	0.04	0.158
	Reduced GHG emissions and climate change	− 0.06	0.085
	Less occupational risk	− 0.08	0.619
	New rural jobs	0.02	0.068
	Reduced fuel import dependency	− 0.13	0.071
	*Expected benefits of GE algae biofuel compared to natural strains*		
	Improve productivity	0.01	0.598
	Require less energy	− *0.03*	*0.021*
	Need less nutrients uptake	0.06	0.696
	Need less use of fresh water	0.03	0.860
	Improve the controllability of the process	− 0.06	0.321
	Improve economic feasibility	− 0.05	0.313
	GMOs should partially replace fossil fuels	− 0.02	0.420
	*Perceptions of social acceptance of GE algae biofuel*		
	Opinion about general social acceptance	− 0.09	0.927
	Variation of public acceptance in case of using gene-editing techniques	0.04	0.776
	Opinion about regulating gene-edited organisms as GMOs	0.06	0.216
	*General risk perception of fuels/power sources*		
	Fossil fuels	− 0.08	0.933
	Established biofuels	0.05	0.740
	GE algae biofuel	− 0.07	0.335
	Solar photovoltaic power	0.04	0.637
	Wind power	− 0.15	0.780
	Hydropower	− 0.16	0.086
Experience in algae industry	*Expected benefits of GE algae biofuel compared to established biofuels*		
	Less environmental impact	0	0.927
	Reduced GHG emissions and climate change	− 0.11	0.659
	No competition with food	0.01	0.397
	Superior engine performance	− 0.01	0.624
	New rural jobs	− 0.06	0.828
	Reduced fuel import dependency	0.03	0.727
	*Expected benefits of GE algae biofuel compared to fossil fuels*		
	Less environmental impact	− 0.11	0.560
	Reduced GHG emissions and climate change	− 0.11	0.822
	Less occupational risk	0.04	0.928
	New rural jobs	− 0.01	0.989
	Reduced fuel import dependency	− 0.02	0.977
	*Expected benefits of GE algae biofuel compared to natural strains*		
	Improve productivity	− 0.06	0.416
	Require less energy	0.03	0.488
	Need less nutrients uptake	− 0.04	0.256
	Need less use of fresh water	0.08	0.107
	Improve the controllability of the process	− 0.06	0.953
	Improve economic feasibility	− 0.15	0.174
	GMOs should partially replace fossil fuels	0.04	0.366
	*Perceptions of social acceptance of GE algae biofuel*		
	Opinion about general social acceptance	0	0.657
	Variation of public acceptance in case of using gene-editing techniques	0.02	0.666
	Opinion about regulating gene-edited organisms as GMOs	− 0.02	0.410
	*General risk perception of fuels/power sources*		
	Fossil fuels	− 0.09	0.450
	Established biofuels	− 0.02	0.951
	GE algae biofuel	− 0.02	0.784
	Solar photovoltaic power	− 0.02	0.714
	Wind power	− 0.03	0.808
	Hydropower	− 0.06	0.835

**Table 9:** Chi-square test *p* values between sociodemographic nominal variables (Appendix A: Table 6) and opinion nominal variables (Appendix A: Table 7)

Sociodemographic nominal variables	Opinion nominal variables	Chi^2^*p* value	Fisher’s test *p* value
Gender	*Personal attitude as final consumer*		
	Opinion about being final consumer of GE algae biofuel	0.467	
	Willingness to pay more money if higher engine performances were achieved compared to established biofuels	0.959	
	Willingness to pay more money if environmental advantages were achieved compared to fossil fuels	0.504	
	*Suggestions to improve general social acceptance*		
	Regulations before any genome engineered species is implemented	0.091	0.144
	Higher or same economic benefits than using fossil fuels	0.774	0.824
	Clear evidence of benefits use of genetic markers	0.479	0.516
	Clear communication of risks and benefits of genome engineering technologies	0.983	1.000
	Rigorous risk assessments of GM algae, involving scientists with minimal conflicts of interest, independent peer review, and public participation	0.079	0.122
	Closed production systems with high security standards	0.217	0.268
	Use of genetic markers	0.132	0.193
	Minor survivability compared to natural strains	0.665	0.818
	Use of new precise gene editing tools instead of traditional genome engineering	0.224	0.256
	Regulations before any genome engineered species is implemented	0.055	0.069
Working field	*Personal attitude as final consumer*		
	Opinion about being final consumer of GE algae biofuel	0.910	
	Willingness to pay more money if higher engine performances were achieved compared to established biofuels	0.757	
	Willingness to pay more money if environmental advantages were achieved compared to fossil fuels	0.637	
	*Suggestions to improve general social acceptance*		
	Regulations before any genome engineered species is implemented	0.389	
	Higher or same economic benefits than using fossil fuels	0.375	
	Clear evidence of benefits use of genetic markers	0.184	
	Clear communication of risks and benefits of genome engineering technologies	0.931	
	Rigorous risk assessments of GM algae, involving scientists with minimal conflicts of interest, independent peer review, and public participation	0.922	
	Closed production systems with high security standards	0.753	
	Use of genetic markers	0.900	
	Minor survivability compared to natural strains	0.136	
	Use of new precise gene editing tools instead of traditional genome engineering	0.500	
	Regulations before any genome engineered species is implemented	0.304	

Fisher’s test was done in cases of having two dichotomous categorical variables

**Table 10:** Chi-square test *p* values between sociodemographic ordinal variables (Appendix A: Table 4) and opinion nominal variables (Appendix A: Table 7)

Sociodemographic ordinal variables	Opinion nominal variables	*p* value	Cramer’s V
Age	*Personal attitude as final consumer*		
	Opinion about being final consumer of GE algae biofuel	0.422	
	Willingness to pay more money if higher engine performances were achieved compared to established biofuels	0.512	
	Willingness to pay more money if environmental advantages were achieved compared to fossil fuels	0.724	
	*Suggestions to improve general social acceptance*		
	Regulations before any genome engineered species is implemented	0.152	
	Higher or same economic benefits than using fossil fuels	0.985	
	Clear evidence of benefits use of genetic markers	0.771	
	Clear communication of risks and benefits of genome engineering technologies	0.372	
	Rigorous risk assessments of GM algae, involving scientists with minimal conflicts of interest, independent peer review, and public participation	0.485	
	Closed production systems with high security standards	*0.041*	*0.221*
	Use of genetic markers	0.914	
	Minor survivability compared to natural strains	0.945	
	Use of new precise gene editing tools instead of traditional genome engineering	*0.044*	*0.220*
	Regulations before any genome engineered species is implemented	0.180	
Educational level	*Personal attitude as final consumer*		
	Opinion about being final consumer of GE algae biofuel	*0.037*	*0.251*
	Willingness to pay more money if higher engine performances were achieved compared to established biofuels	0.580	
	Willingness to pay more money if environmental advantages were achieved compared to fossil fuels	0.651	
	*Suggestions to improve general social acceptance*		
	Regulations before any genome engineered species is implemented	0.565	
	Higher or same economic benefits than using fossil fuels	0.188	
	Clear evidence of benefits use of genetic markers	0.325	
	Clear communication of risks and benefits of genome engineering technologies	*0.004*	*0.346*
	Rigorous risk assessments of GM algae, involving scientists with minimal conflicts of interest, independent peer review, and public participation	0.662	
	Closed production systems with high security standards	0.467	
	Use of genetic markers	0.915	
	Minor survivability compared to natural strains	0.045	
	Use of new precise gene editing tools instead of traditional genome engineering	0.855	
	Regulations before any genome engineered species is implemented	0.184	
Experience in algae industry	*Personal attitude as final consumer*		
	Opinion about being final consumer of GE algae biofuel	*0.024*	*0.236*
	Willingness to pay more money if higher engine performances were achieved compared to established biofuels	0.078	–
	Willingness to pay more money if environmental advantages were achieved compared to fossil fuels	0.458	–
	Suggestions to improve general social acceptance		
	Regulations before any genome engineered species is implemented	0.912	
	Higher or same economic benefits than using fossil fuels	0.988	
	Clear evidence of benefits use of genetic markers	0.339	
	Clear communication of risks and benefits of genome engineering technologies	0.822	
	Rigorous risk assessments of GM algae, involving scientists with minimal conflicts of interest, independent peer review, and public participation	0.562	
	Closed production systems with high security standards	0.412	
	Use of genetic markers	0.672	
	Minor survivability compared to natural strains	0.734	
	Use of new precise gene editing tools instead of traditional genome engineering	0.353	
	Regulations before any genome engineered species is implemented	0.749	

Cramer’s V values were calculated just in cases where *p* values < 0.05

**Table 11:** Chi-square test *p* values between sociodemographic nominal variables (Appendix A: Table 6) and opinion ordinal values (Appendix A: Table 6)

Sociodemographic nominal variables	Opinion ordinal variables	*p* value	Cramer’s V
Gender	*Expected benefits of GE algae biofuel compared to established biofuels*		
	Less environmental impact	0.281	
	Reduced GHG emissions and climate change	0.492	
	No competition with food	0.773	
	Superior engine performance	0.778	
	New rural jobs	0.866	
	Reduced fuel import dependency	0.973	
	*Expected benefits of GE algae biofuel compared to fossil fuels*		
	Less environmental impact	0.412	
	Reduced GHG emissions and climate change	0.800	
	Less occupational risk	0.639	
	New rural jobs	0.302	
	Reduced fuel import dependency	0.143	
	*Expected benefits of GE algae biofuel compared to natural strains*		
	Improve productivity	0.299	
	Require less energy	0.413	
	Need less nutrients uptake	0.408	
	Need less use of fresh water	0.863	
	Improve the controllability of the process	0.920	
	Improve economic feasibility	0.686	
	GMOs should partially replace fossil fuels	0.382	
	*Perceptions of social acceptance of GE algae biofuel*		
	Opinion about general social acceptance	0.129	
	Variation of public acceptance in case of using gene-editing techniques	0.177	
	Opinion about regulating gene-edited organisms as GMOs	0.256	
	*General risk perception of fuels/power sources*		
	Fossil fuels	0.770	
	Established biofuels	0.144	
	GE algae biofuel	0.536	
	Solar photovoltaic power	0.358	
	Wind power	0.887	
	Hydropower	*0.034*	*0.285*
Working field	*Expected benefits of GE algae biofuel compared to established biofuels*		
	Less environmental impact	0.926	
	Reduced GHG emissions and climate change	0.993	
	No competition with food	*0.011*	*0.246*
	Superior engine performance	0.822	
	New rural jobs	0.132	
	Reduced fuel import dependency	0.443	
	*Expected benefits of GE algae biofuel compared to fossil fuels*		
	Less environmental impact	0.750	
	Reduced GHG emissions and climate change	0.981	
	Less occupational risk	0.200	
	New rural jobs	0.802	
	Reduced fuel import dependency	0.786	
	*Expected benefits of GE algae biofuel compared to natural strains*		
	Improve productivity	0.276	
	Require less energy	*0.016*	*0.242*
	Need less nutrients uptake	0.138	
	Need less use of fresh water	0.286	
	Improve the controllability of the process	*0.050*	*0.225*
	Improve economic feasibility	0.138	
	GMOs should partially replace fossil fuels	0.634	
	*Perceptions of social acceptance of GE algae biofuel*		
	Opinion about general social acceptance	0.843	
	Variation of public acceptance in case of using gene-editing techniques	0.720	
	Opinion about regulating gene-edited organisms as GMOs	*0.024*	*0.236*
	*General risk perception of fuels/power sources*		
	Fossil fuels	*0.022*	*0.237*
	Established biofuels	0.238	
	GE algae biofuel	0.126	
	Solar photovoltaic power	0.056	0.230
	Wind power	*0.002*	*0.267*
	Hydropower	0.427	

Cramer’s V values were calculated only in cases where *p* values < 0.05

## Abbreviations

TRL: Technology readiness level
GE: Genetically engineered
EROI: Energy return of investment
EU: European Union
NGO: Non-governmental organization
GMO: Genetically modified organism
GM: Genetically modified
